# Cognitive functioning in Deaf children using Cochlear implants

**DOI:** 10.1186/s12887-021-02534-1

**Published:** 2021-02-10

**Authors:** Fidaa Almomani, Murad O. Al-momani, Soha Garadat, Safa Alqudah, Manal Kassab, Shereen Hamadneh, Grant Rauterkus, Richard Gans

**Affiliations:** 1 Department of Rehabilitation Sciences, Faculty of Applied Medical Sciences, Jordan University of Science and Technology, Irbid, 22110 Jordan; 2 Faculty of Medicine, King Saud University, Riyadh, Saudi Arabia; 3 Department of Hearing and Speech Sciences, The University of Jordan, Amman, Jordan; 4 Faculty of Nursing, Jordan University of Science and Technology, Irbid, 22110 Jordan; 5 Associate (Clinical Fellow) in Nursing at University of Technology, Sydney (UTS), Ultimo, Australia; 6 Department of Maternal and Child Health, Nursing School, Al Al Bayt University, Mafraq, Jordan; 7 American Institute of Balance, Clear Water, Pinellas Park, FL USA; 8 Tulane University, New Orleans, LA USA

**Keywords:** Cognitive abilities, cochlear implant, Development

## Abstract

**Background:**

Cognitive abilities like language, memory, reasoning, visualization, and perceptual functioning shape human action and are considered critical to the successful interaction with the environment. Alternatively, hearing loss can disrupt a child’s ability to communicate, and negatively impact cognitive development. Cochlear implants (CI) restore auditory input thereby supporting communication and may enhance cognitive performance. This study compares general cognitive development after cochlear implantation (2017–2019) in two groups of Jordanian children implanted earlier (age:4–6 years, *N* = 22) and later (7–9 years, *N* = 16) to the development of randomly selected normal hearing peers (*N* = 48).

**Design:**

Visualization, reasoning, memory, and attention were assessed using the Leiter-R scale at baseline (before implantation), 8 months and 16 months post implantation for children with hearing loss. Same times of testing (baseline, 8 months and 16 months) were used for normal hearing peers.

**Results:**

Over the 16-month period, the cognitive improvement of 4–6-year-old deaf children was greater than that of their normal hearing peers on the scales of visualization (5.62 vs. 4.40), reasoning (2.53 vs. 2.38) and memory (17.19 vs. 11.67). while the improvement of 7–9-year-old was less major than that of their normal hearing peers on all scales.

**Conclusions:**

These results suggest that CI not only enhances communication skills but may improve cognitive functioning in deaf children. However, the extent of this improvement was dependent on age at intervention; current results demonstrated that the children received CI at young ages had better cognitive improvements.

## Background

During the early years of life, childhood development is rather a dynamic process that reflects the rapid growth of interrelated functioning such as cognitive, physical, and socio-emotional aspects. In general, cognitive abilities such as language, memory, reasoning, visualization, and perceptual functioning are the skills that shape human action and are critical to the successful interaction with the environment [1–3]. In children, these processes occur at a rapid pace providing an important foundation for lifelong progress. In their early years, typically developing children progress from basic cognitive skills to more complex processes. Early assessment of pediatric cognitive abilities may provide a basic understanding of children’s differences in school performance and other everyday settings. Therefore, early detection of high-risk children increases the chance for early interventions which can provide a greater possibility to retain normal function [1, 4, 5].

Several studies have demonstrated that the process of cognitive development can be affected by a number of risk factors including those with heritability and genetic influences as well as other environmental factors experienced during childhood [1, 6–8]. A number of socio-demographic predictors such as age, gender, parents’ level of education and occupation, family income, exposure to nicotine, school setting, eating habits, and residence and culture areas have shown to be correlated to cognitive abilities in eastern and western cultures [4, 9, 10]. These factors usually reflect the parenting practices, amount of cognitive stimulation given for a child, as well as communication skills and experiences of the parents [2, 8, 11, 12].

Cognitive development is a multi-dimensional process that is interrelated to the development of other skills and abilities; a difficulty or delay in one skill or ability may adversely affect overall cognitive development in children [13]. Unfortunately, developmental delay during early childhood may have profound and permanent consequences [14]. One of the crucial domains strongly associated with cognitive deficits is hearing loss. Lack of listening experience to sounds during early development has been reported to have a negative impact on cognitive abilities [15]. Specifically, auditory deprivation has substantial impacts on neurocognitive development in children. In fact, it has been suggested that difficulties in cognitive skills might be associated with an earlier period of auditory deprivation [16]. A limited access to the auditory environment has been shown to be interrelated to cognitive and social development [13, 14].

Generally, a disruption in the auditory input experienced by deaf and hearing-impaired children can impact normal development of cognitive, psychomotor and behavioral aspects and may later lead to alterations in brain programming [8, 17–20]. Hearing loss has been shown to have a negative effect on the cognitive development of children [21, 22]. In line with these observations, children with hearing impairment were reported to have less attention and more behavioral difficulties than their normal-hearing peers [18, 23]. Mitchell and collogues [1996] reported that 71% of the hearing-impaired children scored in the borderline/abnormal range on attention tasks compared to only 9% of their normal-hearing peers who showed a similar difficulty. Additionally, hearing-impaired children are more likely to have lower scores than others in academic performance [24–27]. Therefore, hearing loss has a tremendous negative effect on child’s everyday activities, self-esteem, and personal autonomy.

In general, extensive research efforts have been devoted to understanding hearing and language development in deaf children implanted with CIs. However, little is known about the developmental changes in cognition after implantation. Cognitive abilities refer to a whole set of thinking skills encompassing language, memory, visualization, attention, reasoning, and executive functions. Examples of executive function tasks involve working memory representations, higher order abstraction, problem solving, concept formation etc. [28, 29]. It has been found that the earlier development of hearing loss happens, the greater impact it will have on child’s cognitive development. It has also been found that the earlier detection and intervention of hearing loss, the lesser the ultimate consequences [30–33].

Several factors affect cognitive development and may underlie some of the reported variations in the efficacy of CIs across recipients. This is an important area to investigate in order to evaluate the effect of restoring auditory input on the learning and cognition of prelingually deafened children. Therefore, the current study aimed to examine the effect of auditory deprivation on cognitive abilities such as visualization, reasoning, attention, and memory, and the extent to which these abilities develop after implantation. The contribution of the cochlear implantation to overall cognitive functions was systematically evaluated before and after the CI surgery. It was hypothesized that cochlear implantation at young age would serve to facilitate cognitive development in deaf children.

This study is different from many other studies for several reasons. First, this study investigated cognitive development of children younger than 10 years of age, while others studied older children [15, 26, 34–36], which could highlight the importance of early hearing management on child development. Second, the study assessed a wide range of cognitive abilities including visualization, reasoning, attention, and memory, whereas other studies focused mainly on learning and memory [16, 22, 37, 38]. Third, deaf children were assessed pre- and post-CI in the current research and differences were calculated compared with demographically matching normal hearing participants similar in variables such as age and gender, area of living, family income, type of school, etc.

This design helped us to evaluate the general effect of the auditory intervention on deaf children while minimizing the effects of maturation and training on cognitive functioning. Finally, to our knowledge, this was the first national study in this geographic area [Jordan, Middle East, and the Arab world] and the first study that assessed the impact of the CI on multiple cognitive abilities. To address the questions associated with this research, we used a multidisciplinary approach bringing together specialists from otolaryngology, audiology, and occupational therapy. The majority of previously published research investigated the effect of cochlear implantation on children’s hearing, speech production, speech perception, and spoken language development. Very little is known about the impact of implantations on particular cognitive abilities and the effect they have on language outcomes.

## Methods

### Participants

The study sample consisted of thirty-eight children [17 males and 21 females] with congenital bilateral severe to profound sensorineural hearing loss [participants’ socio-demographic characteristics are presented in Tables 1 and 2]. All children were diagnosed with hearing loss during their first year of life. Another battery of audiological evaluation was done prior to the surgery, which included a comprehensive set of behavioral and physiological measurements. All children were prelingual and none of them currently used hearing aids. All participating children with hearing loss were recruited from the Ear, nose, and throat [ENT] at King Abdullah University Hospital (KAUH) in Jordan and were scheduled to receive unilateral cochlear implantation in the year of 2017. All children who matched the inclusion criteria were enrolled in the study. All participants were followed for 16 months period.

**Table 1:** Socio-demographic and personal data for the Cochlear Implant (CI) group (*N* = 38) and Normal Hearing (NH) group (*N* = 48) and the result of the Chi-square tests to compare between groups

Variable	CI group	NH group	Chi-square Value	***P***-Value
	N (%)	N (%)		
**Age at first testing**				
4–6 years	22 (58)	24 (50)	0.513	0.200
7–9 years	16 (42)	24 (50)		
Mean ± Standard Deviation	6.16 ± 1.9	6.32 ± 1.3		
**Gender**				
Male	17 (44.7)	21 (43.8)	0.008	1.00
Female	21 (55.3)	27 (56.2)		
**Area of living**				
Urban	20 (52.6)	25 (52.0)	0.037	1.00
Rural	18 (47.4)	23 (48.0)		
**School type**				
Public	15 (39.5)	19 (39.6)	0.631	0.50
Private	23 (60.5)	29 (60.4)		
**Students live with both parents**				
Yes	38 (100)	48 (100)		
No	0 (0)	0 (0)		
**Student’s GPA**				
70–79 (Good)	5 (13.2)	6 (12.5)	0.012	0.99
80–89 (Very Good)	23 (60.5)	29 (60.4)		
90–100 (Excellent)	10 (26.3)	13 (27.1)		
**Family yearly income (1 JD = 1.4 U.S.D)**				
< 6000 JD	31 (81.6)	39 (81.25)	0.002	0.97
> 6000 JD	7 (18.4)	9 (18.75)		
**Child takes breakfast at home before going to school**				
No	9 (23.7)	11 (22.9)	0.007	0.93
Yes	29 (76.3)	37 (77.1)		
**Number of sleep hours**				
8–9 h	21 (55.3)	26 (54.17)	0.010	1.00
10–11 h	17 (44.7)	22 (45.83)

**Table 2:** Parents` variables for Cochlear Implant (CI) group (*N* = 38) and Normal Hearing (NH) group and the results of the Chi-square tests for comparing groups

Variable	CI group	NH group	Chi-square Value	***P***- Value
	N (%)	N (%)		
**Mother occupation**				
Not employed	34 (89.5)	43 (89.6)	0.000	1.000
Employed	4 (10.5)	5 (10.4)		
**Mother’s level of education**				
< high school	7 (18.4)	9 (18.75)	0.103	0.991
High school	24 (63.2)	30 (62.50)		
2-years diploma	4 (10.5)	5 (10.42)		
Bachelor	3 (7.9)	4 (8.33)		
**Father occupation**				
Not employed	1 (2.6)	5 (10.42)	0.001	1.000
Employed	37 (97.4)	43 (89.58)		
**Father `s level of education**				
< high school	18 (47.4)	22 (45.84)	0.022	0.999
High school	10 (26.3)	13 (27.08)		
2-years diploma	3 (7.9)	4 (8.33)		
Bachelor	7 (18.4)	9 (18.75)		
**Mother `s smoking**				
No	36 (94.7)	45 (93.75)	1.051	0.400
Yes	2 (5.3)	3 (6.25)		
**Father’s smoking**				
No	21 (55.3)	27 (56.25)	0.038	1.000
Yes	17 (44.7)	21 (43.75)		

Recruitment and baseline testing were performed 1 week prior to the date of the implantation surgery. In addition, a sample of normal hearing [NH] [hearing thresholds on octave frequencies 250–8000 Hz not exceeding15 dB HL] children were recruited from a compiled research project list of typically developing children. The project collected demographic data and tested the cognitive abilities of 434 normally developed children using the Leiter-R scale [Authors, 2017]. From this project sample, fifty-eight children/parents were recruited using a random number table and 48 children/parents agreed to participate.

The sample of normal hearing children included 24 participants aged 4–6 years at first testing and 24 participants aged 7–9 years at first testing. These participants were chosen to be very similar to the deaf group in terms of age and all other important demographic characteristics that have been proven to be related to cognitive functioning. Therefore, there were no statistical differences between groups in term of age and demographic data. The deaf children were divided into two subgroups according to age [chronological age at time of implantations]: ages 4–6 years [*N* = 22], and 7–9 years [*n* = 16]. Similarly, the NH children were also divided into two age groups: 4–6-year-old [*N* = 24], and 7–9-year-old [*N* = 24]. The normal and deaf participants were similar in all groups by age, gender, area of living, school type, status of living with parents, child’s GPA [Grade Point Average], family yearly income, eating breakfast, sleeping hours and parents’ occupation, level of education and smoking status.

All participants in subject and control groups were between 4 to 9 years of age. All participants were reported to be healthy and free of otological and neurological disorders. Children with learning disabilities, visual impairments, intellectual challenges, developmental delay, or those who were born to deaf parents were excluded from this study. Leiter International Performance Scale-Revised (Leiter-R) [39] was used to evaluate the children’s learning and intellectual abilities. The use of human participants in this study was approved by King Abdullah University Hospital Institutional Review Board and the Deanship of Scientific Research at Jordan University of Science and Technology. Written parental consents and approval from the educational authorities were received before carrying out the study.

### Audiological evaluation before and after cochlear implantation

Comprehensive audiological evaluation was conducted on all participants prior to CI surgery. Generally, a set of behavioral and physiological measurements were completed prior to surgery. Ear-specific pure tone thresholds were obtained using a standard procedure, visual reinforcement audiometry, or play audiometry depending on child’s age by a licensed audiologist. Specifically, air conduction [AC] hearing thresholds were measured at octave frequencies between .25 to 8 kHz and for bone conduction [BC] at octave frequencies .5–.4 kHz. The average hearing thresholds were in the range of sever to profound sensorineural hearing loss. All testing was done in a sound treated booth that meets noise reduction standards. Calibrated diagnostic audiometer type GSI AudioStar Pro was used to assess hearing thresholds using inserts ER-3A earphones. Bone conduction thresholds were measured using B71 transducer and was placed on mastoid bone. In addition, otologists conducted OAEs (otoacoustic emissions) test to find out how well is the cochlea is working and click ABR (auditory brainstem response) test to measure the way the child’s hearing nerve responds to different sounds.

Postoperative aided warble-tone thresholds were assessed in a sound field and revealed an average of 35 dB HL for the frequencies 250–8000 Hz. Post cochlear implant aided hearing thresholds was done on average of 6 months post the surgery when a stable map was reached for CI children. A total of 22 participants received their CI in the right ear and 16 children in the left ear. All children used the same cochlear implant device.

The audiologist confirmed that optimum cochlear implant fitting was achieved for each participant, cochlear implant was functioning normally, and all cochlear implant channels were activated. The otologist who performed the surgery confirmed, by X-ray that cochlear implant electrode was properly placed in the cochlea.

### Speech and language assessment before and after CI

Speech and language assessments were performed by a licensed speech-language pathologist. Before surgery all participants were classified as prelingual. All participants used lip-reading and sign language for communication. Auditory verbal therapy and total communication therapies were provided once every week post CI for 1 year. Speech therapist noted moderate improvement [few two-word sentences] in speech and language of 8 participants of those who were implanted before 6 years of age. Other participants, including those older than 6 years, had poor speech and language development. Most participants continued to use sign language and lip reading as a mode for communication. Eight participants were able to use short two-word sentences but continued to rely mainly on their sign language abilities to communicate.

Important to note that, the CI team [Surgeons, audiologists and speech pathologists] discussed all cochlear implant candidates to determine eligibility. The criteria were rather loose but emphasize that the participants should have bilateral severe to profound sensorineural hearing loss, prelingual and younger than 10 years old. There was no mandatory rule of hearing aid use before CI. Recently, however, hearing aids use for a minimum of 6 months with no significant benefits was added as a pre-condition for CI eligibility.

### Assessment tool

The cognitive abilities of visualization, reasoning, memory, and attention were assessed using the Leiter International Performance Scale-Revised (Leiter-R) [39]. The scale was designed specifically for children with impaired hearing, motor function, communicative ability, and those who speak English as a second language. The scale can be used on normal children and adults from age 2 years to age more than 80 years. The Leiter-R composites were chosen because of their psychometric properties and primarily non-verbal nature. They are more conducive to the evaluation of cognitive abilities in individuals from non-Western cultures who do not primarily speak English.

The instrument contains four composites: (1) the visualization composite, with eight subtests of nonverbal intellectual ability, includes figure ground [FG], form completion [FC], matching [M], picture context [PC], classifications [C], design analogies [DA], paper folding [PF], and figure rotation [FR]; (2) the reasoning composite, with two subtests, includes sequential order [SO] and repeated patterns [RP]; (3) the memory composite, with eight subtests, includes associated pairs [AP], delayed pairs [DP], immediate recognition [IR], delayed recognition [DR], forward memory [FM], reverse memory [RM], spatial memory [SM], and visual coding [VC]; and (4) the attention composite, with two subtests, includes attention sustained [AS] and attention divided [AD] [40].

The researchers did not necessarily administer all items of a specific subtest at all ages as suggested by the developer of the Battery. For example, for 6–10 years we can only administered FG, DA, FC, M, SO, RP, PF subtests from the VR battery and AP, IR, FM, AS, RM, VC, SM, DP, DR, and AD subtests from the AM battery. Less number of items can be administered for younger ages and more items for older ages [39].

The Leiter-R composites have internal consistency [Cronbach’s alpha] reliability coefficients ranging from 0.89 to 0.91% for visualization and reasoning and 0.76 to 0.88% for memory and attention [40]. The instrument also shows consistent evidence of validity from content-analysis studies with extensive item analysis data, criterion-related studies with results for classification accuracy in identifying cognitive delay, and in various construct-related study [40]. The psychometric properties of Leiter-R were not investigated in Arabic language because this assessment is administered mainly by gestures and examples/practice. Authors only translated the language of instructions for the battery for Arabic speaking testers/researchers. A raw score for each of the Leiter-R’s four composites was calculated by totaling the scores of the relevant subtests. Raw scores for visualization and reasoning battery were converted to scaled scores as presented in appendix A and for attention and memory battery as presented in Appendix B in the original Leiter-R battery manual [40]. Generally, a higher score indicates better cognitive performance.

### Translation procedure

The language of instructions for the Leiter-R test battery was translated into Arabic. This translation was for the use of Arabic speaking testers/researchers because the Leiter-R is administered mainly by gestures and examples/practice. The translation was performed by five expert bilingual university professors from Jordan, Saudi Arabia, and the United Arab of Emirates, using a backward-forward translation process [41]. The authors used the same method of translating and standardizing as the Lowenstein Occupational Therapy Cognitive Assessment, Infant /Toddler Sensory Profile and Adolescent/Adult Sensory Profile [References blinded]. Discrepancies in the translation of specific terms were discussed until a consensus was reached. After that, a pilot study with 20 normal hearing children was conducted to evaluate the clarity and readability of the initial versions of the translated subscales; the terms were then modified accordingly, and a revised version was administered to 20 other children. Then, the researchers unanimously agreed that no further modification was required. This translated Arabic version of the language of instructions of the instrument was then translated back into English by a bilingual native English and fluent Arabic speaker, who was unfamiliar with the original versions of the tool.

The process of backward translation was evaluated by ten expert bilingual university professors. The scores of the instrument in evaluating the translation, which is different from the subscales’ scores, ranged from 0 [not similar] to 1 [similar]. A cut score of at least 0.80 was identified to assess the adequacy of the Arabic translation, which implies that 80% or more of the evaluators agreed that the backward translated terms had the same meaning as the original terms. A score below 0.80 suggested a possible problem with the translation. After the translation stage was completed and modifications were attended, all translated terms achieved the cut-off score of 0.80.

### Procedure

All data were collected by two rehabilitation therapists using the Leiter-R scale. For consistency in the administration procedure, both the principal investigator and the two therapists separately assessed and scored a group of 20 children [ten males and ten females aged 4–9 years]. The principal investigator tested 6 children and each therapist tested 7 children. Every testing session was videotaped. Each child was tested once and scored by 3 examiners: the principal investigator and the two therapists. They tested one child after another, scored them separately, and compared their scoring until they reached 98% compatibility/agreement.

All children participating in this study were assessed in a quiet environment, which is the research laboratory of the principal investigator located, and very close to the ENT clinic, where the cochlear implantation surgery was performed. Parents were not present during the testing. All children ate their breakfast before testing. The test was administered between 9 and 11 am to ensure consistency among all participants. The duration of the test ranged from 60 to 90 min [M = 75, SD = 12.4]. All children were offered small incentives such as stickers and smiley faces to keep them attentive and motivated throughout the session. A 15-min break was provided for all children at the middle of the assessment session. The same testing conditions/procedures were used during baseline assessment and re-assessments.

The instructions of the assessment [Leiter-R] were provided pictorially and with visual cues so that children understood the task and were engaged throughout the assessment. Instructions were given in the same way for all participants in all groups. Tests were always administered in the same order, as recommended by the instrument developer, to ensure that the potential effect of the order was the same in both groups. The assessment was administered for the CI group preoperatively [baseline; not more than 1 week before cochlear implant surgery] and then at eight and 16 months post cochlear implant surgery. For the NH participants, a baseline test was administered for each child, and subsequent tests were administered eight and 16 months following the baseline assessment.

### Data analysis

Data were analyzed using the Statistical Package for Social Sciences [SPSS] software version 20.0 [IBM Corp., Armonk, N.Y., USA]. After the cleaning and coding process, descriptive statistics [Means = M and Standard Deviations = SD] were calculated to describe the participants’ cognitive abilities [visualization, reasoning, memory, and attention] using the battery. Descriptive statistics also were calculated to characterize participants’ socio-demographic characteristics. The normality of the data was tested using the Shapiro-Wilks test (*p* < 0.05). The chi-square tests were used to compare the demographic data and the parents’ information between NH group and CI group. A factorial repeated measures analysis of variance [ANOVA] with one factor of age [4–6 years and 7–9 years] and the other factor of hearing status [NH and CI] was performed with time interval [baseline, 8 months, and 16 months]. Separate analyses were conducted for each of four sets of cognitive abilities: visualization, reasoning, memory and attention. Then, multiple comparisons were used between time points; α level was set at 0.05.

## Results

### Demographic results

As shown in Table 1, almost half of the participants were girls [55.3%] and living in urban areas [52.6%] and more than half of them [60.55%] were enrolled in private schools. There were statistical associations in demographics between CI and NH groups as measured by running Chi-square tests for comparing groups [*P* > 0.05].

Additionally, all participating children in the current study resided with both parents [100%], and 81.6% of parents reported a family income of less than 6000 Jordan Dinar per year. The average annual family income of Jordanian families was 3663 Jordan Dinar [JD] [1JD =1.4 USD]. Some children [60.5%] reported a GPA [grade point average] in the school of 80–89% [very good]. In Jordanian elementary schools, the GPA scores ranged from [35–100%]: a score of less than 50% is considered failing and a score of 90–100% is considered excellent. Furthermore, 5.3% of mothers and 44.7% of fathers were smokers [defined as having smoked more than ten cigarettes/day indoors for more than 5 years]. There were statistical associations in parents’ information between NH group and CI group as measured by running the Chi-square tests [*P* > 0.05].

The normal and deaf participants were similar in all groups by age, gender, area of living, school type, status of living with parents, child’s GPA [Grade Point Average], family yearly income, eating breakfast, sleeping hours and parents’ occupation, level of education and smoking status. Authors decided to choose the groups to be similar by these variables because previous research has shown that children’s cognitive abilities are influenced by these factors [1, 4, 6, 8, 9] .

Furthermore, NH group children were not receiving any special educational programs and CI group children were only receiving regular speech therapy sessions at the same center.

### Cognitive results

**Fig. 1:**
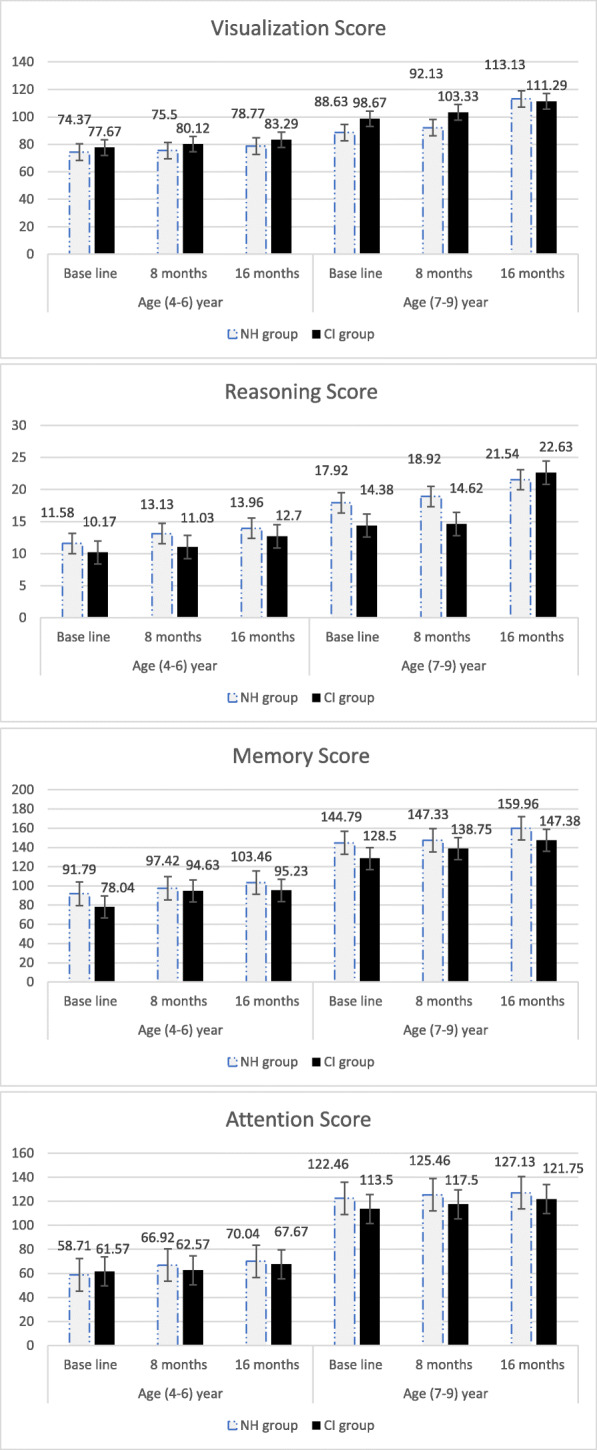
Comparison between Normal Hearing (NH) group (*N* = 48) and Cochlear Implant (CI) group (*N* = 38) across the three-time intervals in Leiter-R subscales

Descriptive statistics by Means and Standard Deviations [SD] for Leiter-R subscales by times of testing and child age for NH group and CI group are reported in detail in Table 3 and presented in Fig. 1. Results show that the two age groups of pre-CI children achieved higher scores on the visualization subtest than the NH children; this was true for the 4–6 year and the 7–9 year age groups, across all time- points On the other hand, scores for the reasoning subscales were lower for the 4–6 year and 7–9 year CI children than those for the 4–6 and 7–9 NH children, across all times of testing. Similarly, the two CI groups performed poorer on the memory subscale than the NH children in the 4–6 year and the 7–9-year age groups, across all times of testing. However, the two age groups with CI and normal hearing children performed almost similarly on the attention measure, across all times of testing.

**Table 3:** Descriptive Statistics by Mean and Standard Deviation (SD) for Leiter-R subscales by time of testing and child Age

Visualization							
**Time of Testing**	**Age**	**NH Group**			**CI Group**		
		N	Mean	SD	N	Mean	SD
Base line Testing	(4–6) year	24	74.37	7.81	22	77.67	10.09
	(7–9) year	24	88.63	6.08	16	98.67	4.81
	Total	48	77.37	12.67	38	88.17	10.91
8 Months Testing	(4–6) year	24	75.50	10.72	22	80.12	10.10
	(7–9) year	24	92.13	5.52	16	103.33	3.09
	Total	48	79.00	14.45	38	91.73	11.35
16 Months Testing	(4–6) year	24	78.77	13.00	22	83.29	6.84
	(7–9) year	24	113.13	7.30	16	111.29	16.81
	Total	48	86.00	17.58	38	97.29	17.08
**Reasoning**							
Time of Testing	**Age**	**NH Group**			**CI Group**		
		N	Mean	SD	N	Mean	SD
Base line Testing	**(4–6) year**	24	11.58	3.15	22	10.17	2.42
	**(7–9) year**	24	17.92	3.31	16	14.38	4.37
	Total	48	14.75	4.52	38	11.11	3.41
8 Months Testing	**(4–6) year**	24	13.13	4.35	22	11.03	3.25
	**(7–9) year**	24	18.92	3.40	16	14.62	4.90
	Total	48	16.02	4.84	38	11.74	3.84
16 Months Testing	**(4–6) year**	24	13.96	4.53	22	12.70	2.96
	**(7–9) year**	24	21.54	3.59	16	22.63	14.88
	Total	48	17.75	5.57	38	14.79	8.10
**Memory**							
Time of Testing	**Age**	**NH Group**			**CI Group**		
		N	Mean	SD	N	Mean	SD
Base line Testing	**(4–6) year**	24	91.79	12.34	22	78.04	12.76
	**(7–9) year**	24	144.79	22.22	16	128.50	23.74
	Total	48	118.29	32.15	38	89.25	26.29
8 Months Testing	**(4–6) year**	24	97.42	14.13	22	94.63	11.75
	**(7–9) year**	24	147.33	23.75	16	138.75	21.14
	Total	48	122.37	31.78	38	103.92	22.91
16 Months Testing	**(4–6) year**	24	103.46	17.92	22	95.23	17.39
	**(7–9) year**	24	159.96	22.79	16	147.38	24.17
	Total	48	131.71	35.02	38	106.21	28.49
**Attention**							
Time of Testing	**Age**	**NH Group**			**CI Group**		
		N	Mean	SD	N	Mean	SD
Base line Testing	**(4–6) year**	24	58.71	15.28	22	61.57	11.92
	**(7–9) year**	24	122.46	23.27	16	113.50	25.09
	Total	48	90.58	37.64	38	89.04	18.51
8 Months Testing	**(4–6) year**	24	66.92	14.17	22	62.57	17.74
	**(7–9) year**	24	125.46	22.97	16	117.50	24.08
	Total	48	96.19	35.09	38	90.04	20.91
16 Months Testing	**(4–6) year**	24	70.04	18.22	22	67.67	11.94
	**(7–9) year**	24	127.13	22.80	16	121.75	23.61
	Total	48	98.58	35.34	38	79.05	26.77

Table 4 shows the results of the factorial repeated measures ANOVA for Leiter-R subscales with interactions by times of testing [baseline, 8 months, 16 months] and child age group [[4-6 years], [7–9 years]] and by times of testing [baseline, 8 months, 16 months] and child hearing status [NH group, CI group]. Visualization scores improved significantly for NH group across times of testing [*F* = 8.687, *P* = 0.000], and improved significantly more for [4–6 years] age group [*F* = 28.530, *P* = 0.000]. On the other hands, reasoning scores improved significantly across times of testing for CI group [*F* = 3.278, *P* = 0.042] and for both age groups; [4–6 years, *F* = 5.972, *P* = 0.004] and [7–9 years, *F* = 4.056, *P* = 0.019].

**Table 4:** Factorial repeated measures ANOVA for Leiter-R subscales with interaction by times of testing and child age group and by times of testing and child hearing status (Normal Hearing (NH) Group or Cochlear Implant (CI) Group)

Visualization				
**Source**	**NH Group**		**CH Group**	
	F	P.	F	P.
Time of Testing	17.343	0.000	13.080	0.000
Age Group	102.136	0.000	268.721	0.000
Time of Testing Age Group	8.687	0.000	1.980	0.142
**Source**	**Age (4–6) year**		**Age (7–9) year**	
	F	P.	F	P.
Time of Testing	18.336	0.000	1.242	0.292
Hearing Status	13.612	0.000	7.070	0.009
Time of Testing Hearing Status	28.530	0.000	2.362	0.098
**Reasoning**				
**Source**	**NH Group**		**CI Group**	
	F	P.	F	P.
Time of Testing	7.706	.001	9.228	0.000
Age Group	110.006	.000	27.655	0.000
Time of Testing Age Group	0.717	.490	3.278	0.042
**Source**	**Age (4–6) year**		**Age (7–9) year**	
	F	P.	F	P.
Time of Testing	4.372	0.015	2.871	0.060
Hearing Status	3.077	0.083	8.438	0.004
Time of Testing Hearing Status	5.972	0.004	4.059	0.019
**Memory**				
**Source**	**NH Group**		**CI Group**	
	F	P.	F	P.
Time of Testing	6.053	0.003	8.221	0.000
Age Group	271.037	0.000	168.713	0.000
Time of Testing Age Group	0.347	0.707	0.423	0.656
**Source**	**Age (4–6) year**		**Age (7–9) year**	
	F	P.	F	P.
Time of Testing	13.859	0.000	2.126	0.125
Hearing Status	12.721	0.000	5.324	0.023
Time of Testing Hearing Status	2.091	0.127	1.409	0.250
**Attention**				
**Source**	**NH Group**		**CI Group**	
	F	P.	F	P.
Time of Testing	2.061	0.131	1.125	0.329
Age Group	327.870	0.000	197.949	0.000
Time of Testing Age Group	0.375	0.688	0.147	0.864
**Source**	**Age (4–6) year**		**Age (7–9) year**	
	F	P.	F	P.
Time of Testing	4.031	0.020	0.462	0.632
Hearing Status	0.296	0.587	1.829	0.180
Time of Testing Hearing Status	1.312	0.272	0.038	0.963

Memory scores were improved for both groups across times of testing; however, the scores’ improvements were higher for children in CI group [*F* = 8.221, *P* = 0.000] than children in NH group [*F* = 6.053, *P* = 0.003] and were the highest in [4–6] age in CI group [*F* = 12.721, P = 0.000]. Attention scores were not improved significantly across times of testing in both age groups; see more details on Table 4.

Multiple comparisons using the LSD [Least Significant Difference] test was performed for Leiter-R subscales between times of testing for NH and CI groups. The results showed that performance on visualization [MD = 8.63, 9.13; *P* = 0.000, 0.000], reasoning [MD = 3.00, 3.78; P = 0.000, 0.000] and memory [MD = 13.42, 16.96; *P* = 0.001, 0.000] subscales improved significantly for NH and CI groups, respectively, after 16 months when compared to the baseline which is consistent with maturation. However, the mean differences for the CI group were higher than the NH group. Moreover, for 8 to 16 months interval, the only significant differences were seen for CI group in reasoning [MD = 0.73, P = 0.000] and memory [MD = 3.05, *P* = 0.008] scores, see Table 5 for more details.

**Table 5:** Multiple Comparisons for Leiter-R subscales between times of testing using LSD test for Normal Hearing (NH) group and Cochlear Implant (CI) group

Visualization					
		**NH Group**		**CI Group**	
(I) Time of Testing	(J) Time of Testing	Mean Difference (I-J)	*P* value	Mean Difference (I-J)	*P* value
Base line Testing	8 Months Testing	1.63	0.449	3.56	0.040
	16 Months Testing	8.63	0.000	9.13	0.000
8 Months Testing	16 Months Testing	5.00	0.002	5.56	0.002
**Reasoning**					
		**NH Group**		**CI Group**	
(I) Time of Testing	(J) Time of Testing	Mean Difference (I-J)	*P* value	Mean Difference (I-J)	*P* value
Base line Testing	8 Months Testing	1.27	0.100	0.630	0.575
	16 Months Testing	3.00	0.000	3.78	0.000
8 Months Testing	16 Months Testing	0.73	0.226	3.05	0.008
**Memory**					
		**NH Group**		**CI Group**	
(I) time of testing	(J) time of testing	Mean Difference (I-J)	*P* value	Mean Difference (I-J)	*P* value
Base line testing	8 months testing	4.08	0.303	14.67	0.000
	16 months testing	13.42	0.001	16.96	0.000
8 Months testing	16 months testing	2.29	0.543	9.33	0.020
**Attention**					
		**NH Group**		**CI Group**	
(I) time of testing	(J) time of testing	Mean Difference (I-J)	*P* value	Mean Difference (I-J)	*P* value
Base line Testing	8 Months Testing	5.60	0.168		
	16 Months Testing	8.00	0.060		
8 Months Testing	16 Months Testing	2.40	0.555		

## Discussion

This work investigated the effect of cochlear implantation on cognitive functioning in prelingually [before language acquisition] deafened children. This study expands our understanding of the CI effect by comparing the development of a variety of cognitive abilities before and after cochlear implantation in prelingually deafened children in two different age ranges. The current study addressed a number of critical phenomena. Comparing groups younger than 10 years of age allows us to address the importance of early hearing intervention in many types of development. Considering a wide range of cognitive abilities [visualization, reasoning, attention, and memory] allows us to better illustrate the developmental significance of auditory input over time. Thus, we are able to track the effects of cochlear implantation and to investigate the resulting developmental trajectory of cognitive functioning.

Early exposure to auditory sounds is crucial for auditory development [23, 42]. This early auditory experience is particularly important, as neural plasticity peaks during the first few years of life [43]. In fact, a peak of cortical activity has been found at the age of four, which significantly contributes to child language and cognitive development [44]. It follows that children who receive their CI before 27 months develop cognitive skills comparable to that in their NH peers [45]. Generally, the literature shows a positive correlation between outcome measures and age at implantation [15, 46]. In the current study, children who were implanted between ages 4 and 6 demonstrated higher rates of cognitive improvement, especially in memory and attention subscales, than their normal hearing peers, while 7–9-year-old only kept pace developmentally with their normal hearing peers.

Some work has shown that children with CIs are outperformed by age matched NH peers in cognitive tasks that rely on phonological processing [47]. While variance due to age at the time of assessment, time of implantation, and maternal education levels may complicate the conclusions that can be drawn about the differences in performance of some cognitive skills, children with CIs steadily develop after implantation at magnitudes well below the development of age-matched peers 38]. These trends in development after implantation affirm the importance of early intervention that results in natural promotion of social, phonological, perceptual, and cognitive development. The Leiter-R scales, designed specifically to assess non-verbal cognition, demonstrated that post-implantation development of a transcendent range of cognitive skills is dependent upon the age auditory input is established in the child’s brain. Early implantation and auditory rehabilitation are critical, as compromised development in these areas ultimately adversely affects quality of life of deaf children.

This study demonstrates the compromising nature of the absence of auditory input to cognitive development over prolonged amounts of time. Prior to implantation, deaf children exhibited lower scores on memory and reasoning subtests compared to their NH peers. This indicates that adequate auditory input is necessary in order to develop and support these forms of cognition. These results are consistent with existing literature [47–49] The finding that young children [4–6 years] with CIs and their NH peer had similar performance improvements on the attention subtests is supported by some previous research [27, 33, 50, 51]. Attention in deaf individuals is perhaps mediated by non-auditory stimuli like visual cues.

Further, the results showed that deaf children outperformed NH children on tests of visualization at baseline. This could suggest a relationship between the development of visualization and attention skills and hearing impairment. It has been suggested that deaf children develop visual-spatial advantages and enhanced visual cognition. In fact, they are often described as more heavily relying on visual stimuli and spatial schematics to learn and problem solve than their normal hearing peers [26, 34, 49, 52–55]. To a certain extent, brain plasticity during development can explain [function migration] the development patterns of visual skills in children with hearing loss. This assumption has been supported by neurophysiological and MRI studies [35].

The improvement in Leiter-R scores post cochlear implantation in deaf children on cognitive skills, mainly memory and reasoning, was remarkable. The CI group had higher scores at the 16-month intervals than their baselines, suggesting the importance of auditory input to cognitive functioning. The same instrument [Leiter-R] has demonstrated that nonverbal, cognitive skills improve after cochlear implantation [16, 56–59]. For example, a neuropsychological battery was administered to 17 deaf children [mean age 7 years] before implantation and 6 months after implantation; while the children showed marked improvements in nonverbal cognitive abilities, their performance in verbal skills did not change [59]. In contrast to the CI group, NH children’s scores on reasoning and memory were not significantly different 8 months after baseline testing. They did, however, achieve better scores 16 months after baseline testing. These results likely reflect the trajectories of cognitive abilities in typically developing children.

With increased experience with cochlear implants, attention skills continued to improve in the CI group. It has been hypothesized that improvement in attention capacity after cochlear implantation might lead to overall improvement in cognitive functioning [57]; this notion ought to be further investigated. It is likely that many children are enrolled in aural habilitation therapy following implantation. Improvement in cognitive abilities may reflect more accurately the cumulative effects of auditory training and speech therapy. Auditory training and speech therapy positively affects problem-solving skills and other cognitive functioning [56]. Therapists may consider the earliest cochlear implantation in conjunction with speech and language therapy for individuals with hearing loss/impairments. This approach may promote faster cognitive development. Further investigation is necessary to maximize the benefits of cochlear implantation rehabilitation in deaf individuals.

Interestingly, over the study period, the amount of demonstrated improvement on the subtests was greater for the CI group than the NH group, especially in the reasoning and memory subscales. This difference in improvement, however, was observed in the younger CI children [4–6 years], especially in the memory and attention subscales. The rates of improvement in Leiter-R scores were not statistically significantly different between older CI children and their matched NH peers. This suggests that these two groups of children developed at a similar rate, implying that the age of implantation is critical in defining the overall outcome of the implantation. Age of implantation along with a variety of other demographic factors [chronological age, duration of deafness] influence cognitive development subsequent to implantation [15, 38, 46, 59–61]. However, the differences for 7–9-year-old were not statistically significant [*P* > 0.05]; this might be related to the relatively small *sample size* in this study. Therefore, results from the current data should be interpreted carefully.

This study addresses the impact of cochlear implantation on cognition and cognitive development by assessing visualization, attention, reasoning, and memory in children who were recently implanted. Current findings suggest that cochlear implantation can improve cognitive performance and development by restoring auditory input. The impacts of visual memory skills and attention on cognition ought to be the participant of further research in order to better understand the context in which auditory input supports overall cognitive development. Cochlear implants are incredibly effective in restoring hearing to profoundly deaf and severely hearing-impaired individuals. Cochlear implantation leads to improved quality of life for a large number of recipients worldwide [62–65].

### Limitations

The study had some limitations. First, the average age of cochlear implantation in our study is high [6.16] years. It was found that early implanted children [before the age of 27 months] had comparable cognitive skills to those of normal hearing peers [58]. Second, the type and frequency of different interventions that the cochlear implant children are getting in this study are not available. Therefore, the improvement in cognitive abilities may reflect the cumulative effects of other therapies. Third, the study sample size was rather small, not a population-based sample, done only over a period of 16 months and tested only cognitive abilities. Therefore, future research with larger population-based sample size will be necessary to assess the long-term effect of unilateral and bilateral cochlear implantations on cognitive skills as well as other factors such as motor skills, social activity, quality of life, self-esteem, personal autonomy, etc. Fourth, the effect of psychosocial factors [such as the birth order] that could predict the outcomes of cochlear implantations on cognitive abilities were not examined [55].

## Conclusion

These results suggest that CI not only enhances communication skills but may improve cognitive functioning in deaf children. However, the extent of this improvement was dependent on age at intervention; current results demonstrated that the children received CI at young ages had better cognitive improvements.

## Data Availability

Supporting data can be accessed by emailing the corresponding author at falmomani@just.eu.jo.

## Abbreviations

FG: Figure ground
FC: Form completion
M: Matching
PC: Picture context
C: Classifications
DA: Design analogies
PF: Paper folding
FR: Figure rotation
SO: Sequential order
RP: Repeated patterns
AP: Associated pairs
DP: Delayed pairs
IR: Immediate recognition
DR: Delayed recognition
FM: Forward memory
RM: Reverse memory
SM: Spatial memory
VC: Visual coding
AS: Attention sustained
AD: Attention divided
CI: Cochlear implants
NH: Normal hearing
GPA: Grade point average
OAEs: OtoAcoustic emissions
ABR: Auditory brainstem response
LSD: Least Significant Difference
